# Commencement Atlanta Medical College

**Published:** 1893-03

**Authors:** 


### THIRTY-FIFTH ANNUAL COMMENCEMENT OF THE ATLANTA MEDICAL COLLEGE.

The exercises were held in DeGives Opera House, and were opened with a prayer by the Rev. Dr. Morrison. This was followed by the report of the faculty by the proctor, Dr. W. S. Kendrick. The report was a most interesting one, and was a succinct review of the history of the college. Dr. Kendrick said:

In closing the thirty-fifth annual session of the Atlanta Medical College, the following report is submitted:

Each year brings additional prosperity and success to this well established institution. The faculty, proudly conscious of the growing popularity of this school, has been honored during the past winter with the largest class in the history of the college. With the strongest competition on every side, the school stands without a peer in all this section of the south. The faculty, untiring in effort, and conscientious in the discharge of duty, has been complimented with 172 students during the past session.

Georgia 132, Alabama 12, South Carolina 12, Florida 7, North Carolina 4, Texas 2, Cuba 1, New York 1, Tennessee 1. Total, 172.

IN PHARMACEUTICAL DEPARTMENT:

Georgia 8, South Carolina 2, Alabama 1. Total, 11.

After the report of the proctor, Col. N. J. Hammond, President of the Board of Trustees, conferred the degrees in his usually happy style.

Hon. Benj. J. Conyers, the orator of the evening, came next, and his address was one to which the audience gave its undivided attention.

The valedictorian, Dr. J. M. Thomas, of Atlanta, was the next, and his address was one of the best and most enjoyable the commencement of the College has ever known.

After Dr. Thomas' address, the prizes were delivered by Judge W. R, Hammond. The first prize went to Dr. William Wightman Mangum, of Alabama; the second to Dr. Leroy Campbell, of Georgia. Honorable mention was made of Drs. W. H. Bryan, J. E. Murray, W. D. Hicks, G. L. Alexander, J. C. Mull and J. C. Hooten.

THE GRADUATING CLASS.

G. L. Alexander, O. R. Alexander, W. T. Asher, F. O. Auxford, A. L. R. Avant, J. H. Bradfield, R. L. Breedlove, J. L. Copeland, G. M. Crawford, M. D. Cunningham, V. R. Dallas, F. Davis, J. H. Dennard, Wiley Fussell, N. W. Gable, H. W. Gibney, J. M. Gregory, R. L. Grier, J. I. Griffeth, J. B. Gurley, W. M. Johnson, J. H. Lewis, L. V. B. McCormick, H. E. McDowell, J. L. McLean, W. W. Mangum, I. W. Martin, M. V. Miller, J. R. Sewell, Jr., T. B. Sheppard, R. N. Spinks, M. N. Stow, J. N. Tanlinson, T. J. Taylor, G. R. Thigpen, H. W. Thomas, W. H. Bryan, J. R. Russell, J. L. Campbell, C. H. Carroll, A. J. Cavendar, J. E. Christian, H. L. Cook, E. W. Dilbon, J. B. Douglass, T. J. Dunlop, R. B. Durrett, B. F. Eaves, W. S. Emery, T. J. Fales, H. E. Felton, W. D. Hicks, J. C. Hooten, E. B. Howell, W. S. Howell, J. S. Humphreys, O. L. P. Jackson, W. L. Jerkins, S. M. Johnson. Elijah Morgan, R. W. J. Newman, W. E. Pate, O. N. Pendergrass, L. E. Roper, J. M. Thomas, W. J. Turner, C. J. Vaughan, J. S. Vaughan, W. A. Walden, S. D. Warnock, E. M. Wright, H. S. Wright.

GRADUATES IN PHARMACY.

F. A. Ingraham, C. N. Smith, J. S. Smith, L. P. Youmans.

---

DR. E. C. RIPLEY, of this city, was married to Miss Sarah Houston, of Decatur, Ga., on March 1st. Dr. Ripley is associated with Dr. W. C. Jarnigan, and is one of the rising young physician of this city. The RECORD extends congratulations to the happy couple.

